# Enzymes-assisted green ultrasonic extraction with UPLC-DAD quantification of phenolic compounds in chia seeds with a comparative evaluation based on geographical origins

**DOI:** 10.1016/j.ultsonch.2026.107856

**Published:** 2026-04-15

**Authors:** Rizwan Ahmad, Aljawharah Alqathama, Mohammed Aldholmi, Mohammad Alkawi, Faisal Alnaeem, Majed Khan, Mutaz Algarzai, Mohd Amir, Muhammad Riaz

**Affiliations:** a Department of Natural Products, College of Pharmacy, Imam Abdulrahman Bin Faisal University, Kingdom of Saudi Arabia; b Department of Pharmaceutical Sciences, Faculty of Pharmacy, Umm Al-Qura University, Makkah 21955, Kingdom of Saudi Arabia; c Department of Pharmacy, Shaheed Benazir Bhutto University Sheringal, Dir, Upper Khyber Pakhtun Khwa, Pakistan

**Keywords:** Chia seeds, Green-ultrasound extraction, Enzymes models, UPLC-DAD, Phenolic

## Abstract

**Background:**

This study aims to enhance the recovery of chia phenolic using a green ultrasound extraction (UA) with different individual and mixed-enzymes models followed by a simultaneous determination of the phenolic [chlorogenic acid (cGA), rosmarinic acid (RA), ferulic acid (FA), quercetin (QT), kaempferol (KF)] using an in-house developed UPLC-DAD method across eleven different geographical origins.

**Methodology:**

Green solvents of acetone (ACT), ethanol (EtOH), and water (H_2_O) were used for UA of chia phenolic whereas, UPLC-DAD method was validated for simultaneous determination of phenolic. The enzymes of protease, cellulase, viscozyme, and α-amylase (1–5%) were applied to enhance the phenolic recovery.

**Results:**

The optimal solvent (UA-MD) with highest phenolic recovery (168.41 ppm) was ACT: H_2_O (70: 30 v/v) whereas, UA-MV revealed the order for phenolic yield: US > KSA > Ecuador. The pretreatment with enzymes resulted more yield for RA, cGA, and FA using viscozyme and α-amylase whereas, protease enzyme favored QT and KF. The mixed enzymes model showed synergistic effects for protease–amylase and cellulase–viscozyme systems. The UPLC-DAD method exhibited a separation in 8 min with excellent sensitivity LOD (0.02–0.14 ppm).

**Conclusion:**

An effective green UA-based enzymes assisted model was developed with an enhanced release for chia phenolic.

## 1 Introduction

Chia seeds obtained from *Salvia hispanica*, is a plant native to Guatemala and Mexico. These seeds are consumed as food and has importance since ancient times [1]. Based on their importance, chia seeds are cultivated and distributed worldwide. According to FDA, United states of America (US) recognized chia seeds as safe whereas, European Union recognized it as a novel food in 2009 with a widespread consumption in countries such as Australia [1].

The importance as functional food, chia seeds gained considerable popularity where the health promoting properties are commonly ascribed to their rich biochemical composition [2]. Chia seeds contain substantial amounts of protein, omega-3 fatty acids, dietary fiber, vitamins, minerals, and a diverse range of polyphenolic compounds [3]. Among these constituents, polyphenolics are particularly associated with functional and pharmacological effects. The reported compounds include phenolic acids such as chlorogenic acid, rosmarinic acid, caffeic acid, gallic acid, ferulic acid, and salvianolic acid, as well as flavonoids including daidzein, kaempferol, and quercetin [4], [5], [6]. The increasing evidence suggests that a notable fraction of chia phenolic may occur in bound or matrix-associated forms rather than as freely extractable molecules, linked to structural components of the seed matrix, which can restrict their release under conventional extraction conditions. [4], [7], [8]. Current scenario is particularly relevant for chia seeds because of their complex structural characteristics. Chia seeds are rich in dietary fiber, mucilage, proteins, and polysaccharides. Upon hydration, these components can swell and form a viscous matrix that limits solvent penetration and slows mass transfer results in incomplete phenolic even with the use of effective solvents [4], [7], [9], [10].

The phytochemical composition of chia seeds varies according to climate, soil composition, cultivar, processing conditions, and extraction techniques. Previous studies reported extractions from chia seeds however, the extraction approaches employed [11], [12], present differences in reported phenolic contents. This reflect not only biological variation but also methodological limitations, particularly the limited ability of conventional extraction procedures to disrupt the chia seed matrix and release bound phenolic fractions.

To address these limitations, current study aims to employ enzyme-assisted extraction for improving phenolic recovery. The enzymes such as cellulase, amylase, protease, and multi-enzyme preparations can degrade specific matrix components that have entrapped bioactive compounds facilitating more effective release of phenolic from complex seed matrices [8], [13]. A systematic comparisons of individual and combined enzyme systems for chia seeds phenolic remain to be explored [7], [14], [15].

The phytochemical profiles of chia seeds present a wide variation due to differences in geographical regions [16], mostly due to variations in climate, soil composition, agricultural practices, and post-harvest handling. For instance, the germination and solid state fermentation processes enhances the total phenolic content and antioxidant activities of chia seeds., as reported [17], [18]. This urge for a standardized extraction and quantification strategy that can support meaningful quality evaluation.

Furthermore, the paradigm-shift towards environmentally safer and sustainable approaches the exploration of green extraction methods is the needed. Ultrasonic-assisted extraction enhances phytochemical recovery primarily through acoustic cavitation, which promotes micro-scale cell disruption, improves solvent penetration, and accelerates mass transfer. The use of appropriate green solvent systems and enzymatic pretreatment, may further enhance the release of phenolic from structurally complex matrices such as chia seeds. Although ultrasonic-assisted extraction has previously been applied to chia seeds [10], earlier studies relied on non-green solvents, including methanol and n-hexane, and did not report phenolic quantification using advanced hyphenated analytical techniques.

The reliable evaluation of extraction performance depends on the analytical method employed. The simultaneous determination of multiple phenolic acids and flavonoids, often present at markedly different concentration levels, requires analytical techniques that offer high resolution, sensitivity, and speed. Ultra-performance liquid chromatography coupled with diode array detection (UPLC-DAD) provides clear advantages over conventional HPLC in this regard [19], [20].

In this study, a green extraction approach using water, ethanol, and acetone, alone and in combination, is developed and coupled with a fast and reproducible UPLC-DAD method for the simultaneous determination of five phenolic compounds: chlorogenic acid, rosmarinic acid, ferulic acid, quercetin, and kaempferol. The effect of enzymatic treatment on phenolic recovery is also examined. The optimized enzyme-assisted green extraction strategy is subsequently applied to chia seeds of different geographical origins available in the Saudi market, with the aim of evaluating compositional variability and supporting quality-based standardization.

## 2 Materials and methodology

### 2.1 Instruments, solvents, and chemicals

Enzymes: α-amylase from Aspergillus oryzae, cellulase from *Trichoderma reesei*, and viscozyme V2010 [Sigma Aldrich St Louis, MO, U.S.A.], protease from Aspergillus oryzae [UFCBio, NY, U.S.A]; Standard drugs: chlorogenic acid (cGA) [UFCBio, NY, U.S.A], rosmarinic acid (RA), ferulic acid (FA), quercetin (QT), kaempferol (KF) [Sigma Aldrich St Louis, MO, U.S.A.]; HPLC- and analytical-grade solvents of acetone (AC), ethanol (EtOH), acetonitrile (ACN) [Merck Darmstadt, Germany]; whereas, distilled water (H2O) [Millipore, USA]; For extraction of samples: high frequency 20 kHz ultrasonic dismembrator with a probe and transducer [Fisher Scientific]; For quantification of samples: UPLC with DAD (diode array detector) [Shimadzu, Japan].

### 2.2 Collection and processing of the chia samples

This study recruited eleven different origins sample for chia seeds throughout the Kingdom using the accessible online platforms as well as in-person visits to various local malls and grocery stores. The pre-packed and sealed samples were transferred to the lab and coded for ease of identification: Saudi Arabia (C1), Ecuador (C2), Bahrain (C3), India (C4), Argentina (C5), Mexico (C6), yellowish Peru seeds (C7), brownish Peru seeds (C8), Bolivia (C9), United States of America (C10), and Spain (C11). All the samples were stored in the refrigerator at a temperature of 4°C, till further processing. For all samples of chia seeds, storage in airtight containers at 4°C in the dark was employed before analysis to prevent degradation of phenolic compounds. Since all samples of chia seeds were obtained from commercial suppliers i.e., local malls and herbal shops with different geographical origins, detailed agronomic data for samples such as cultivar, time of harvest, and environmental conditions were not available. However, all samples of chia seeds were treated in exactly the same way before analysis.

### 2.3 Ultrasound assisted (UA) green extraction for phenolic

#### 2.3.1 Extraction method development (UA-MD)

The phenolic from chia seeds were extracted with the help of ultra sound assisted extraction (UA) using the three different green solvents of acetone (AC), ethanol (EtOH), and water (H_2_O). The parameters for UA extraction were adopted from the previously reported study [21], with slight modifications: amplitude (40%), pulse (10 s/5s), extraction time (5 min), sample amount (100 mg), and solvent volume (10 mL). The extraction model consisted of twelve samples for the three solvents and their mixtures in different ratio: 1; AC (100%), 2; EtOH (100%), 3; H_2_O (100%), 4; AC: EtOH (50:50%), 5; AC: EtOH (30:70%), 6; AC: EtOH (70:30%), 7; AC: H_2_O (50: 50%), 8; AC: H_2_O (30:70%), 9; AC: H_2_O (70:30%), 10; EtOH: H_2_O (50:50%), 11; EtOH: H_2_O (30:70%), and 12; AC: H_2_O (70:30%). The extracted samples were filtered (20 mm cellulose filter paper), centrifuged (13000 rpm, 10 min), and diluted for UPLC analysis of the phenolic: cGA, RA, FA, QT, and KF. For UPLC analysis, all the diluted sample were dissolved in HPLC-grade solvents and syringe-filtered (0.2μ). All the experiments were performed in triplicate (*n = 3*) and results are presented as mean ± standard deviation.

#### 2.3.2 Extraction method validation (UA-MV)

The solvent system with more yield for the phenolic amount in the chia seeds sample was selected and the UA extraction method was validated on a small scale where chia seeds were extracted in large amount. In detail: 10 g of the eleven different geographical origins chia seeds samples (C1-C11) were extracted in 60 mL of the solvent using UA technique, filtered (20 mm cellulose filter paper), dried using Genevac, and the extract yields were calculated. The extraction parameters for UA-MV remain alike UA-MD. For comparative analysis of the phenolic in the eleven samples, the dried samples were re-dissolved in HPLC-grade solvents, diluted at the desired concentrations, syringe-filtered (0.2μ), and subjected to UPLC analysis.

### 2.4 UPLC analysis for chia phenolic

#### 2.4.1 Phenolic standard and stock solutions

The standard drugs for cGA, RA, FA, QT, and KF were dissolved (1 mg/1mL) in HPLC-grade EtOH: ACN (1:1 mixture) to prepare standard solutions. An intermediate mix-standards stock solution was prepared for working to prepare the calibration curve (CC) points for the phenolic-mix. The working standard was diluted in required concentrations to prepare the CC-points in the linearity range of: 5–200 ppm (5, 10, 50, 100, 200) for cGA and RA, and 1–100 ppm (1, 5, 10, 50, 100) for FA, QT, and KF standard drugs. The final CC-points solutions were diluted in HPLC-grade ACN, syringe-filtered, coded, and analyzed for constructing the CC. All the experiments were performed in triplicate (*n = 3*) and results are presented as mean ± standard deviation.

#### 2.4.2 UPLC analytical method development

To develop optimal chromatographic conditions for the phenolic, a set of mobile phases (EtOH: H_2_O, MeOH: H_2_O, and ACN: H_2_O) with varying concentrations (5–95% B) of isocratic and gradient flow, different injection volumes (1–10 μ L), column oven temperatures (30-40°C), mobile phase flow rates (0.1–0.7 mL/min), mobile phase additives (formic acid; FA) in concentrations range of 0.1–1%, and columns with different specifications (C8 and C18) were evaluated. To produce the appropriate intensities for phenolic-peaks and, preserve the peak shapes and smoothness in the chromatogram, the concentrations for the sample were varied from 5 ppm-200 ppm. All the experiments were performed in triplicate (*n = 3*) and results are presented as mean ± standard deviation.

#### 2.4.3 UPLC analytical method validation

The developed UPLC analytical method for chia seeds phenolic was validated according the ICH (International Council for Harmonization) guidelines, as reported [22], The method was validated by assessing its specificity, accuracy, linearity, peak resolution (Rs), limit of detection (LOD), and limit of quantification (LOQ). To confirm the specificity, blank samples were analyzed under the established chromatographic conditions where no peaks were observed at the retention times corresponding to the phenolic compounds, demonstrating the method’s selectivity. For the linearity, the method was assessed using five different calibration points were prepared in the range of 5–200 ppm for cGA and RA, and 1–100 ppm for FA, QT, and KF. The linearity range for pharmaceuticals in the range of r^2^ > 0.995 was assessed. The accuracy, LOD, and LOQ were calculated based on signal-to-noise ratios of 3 and 10, respectively. The data for peak resolutions (Rs) and the percentage relative standard deviation (%RSD) for the phenolic compounds were noted.

### 2.5 Phenolic extraction using enzymes models (EM)

#### 2.5.1 Individual enzymes solutions preparation

The initial phase for the enzymatic study prepared different concentrations for the four different types of enzymes namely: protease, cellulase, viscozyme, and α-amylase. The concentrations (w/v) used for the four enzymes consisted of 1%, 2.5%, and 5%. In detail, 1 g in 100 mL of distilled water was dissolved for the enzymes to prepare 1% stock solution whereas, 2.5 g and 5 g in 100 mL distilled water were used to prepare 2% and 5% stock solutions for proteases, cellulase, viscozyme, and α-amylase. The solutions were ready to use due to the high water solubility for the enzymes in aqueous solutions. No extra treatment for the vortex, sonication, heating, or filtration was required. The pH for the medium was maintained in the range of 5.5–6.5 throughout the study whereas, the prepared enzymes solutions were placed in refrigerator at 4°C for short term storage. The aim of the study is to developed a novel extraction and analytical method hence, only one factor of the enzymes concentrations (1–5%) was evaluated herein. In order to understand the effect of enzymes on phenolic yield mechanistically, a detailed multifactorial study taking into consideration the effect of pH, temperatures, time duration, and solid-to-solvent ration need to be conducted. All the experiments were performed in triplicate (*n = 3*) and results are presented as mean ± standard deviation

#### 2.5.2 Mix-enzymes model (MEM)

The second phase for enzymatic study used a mix of different enzymes in an equal proportion 1:1 for protease, cellulase, viscozyme, and α-amylase. Briefly: 2.5% (2.5 g in 100 mL of distilled water) of the enzymes concentrations were used to prepare the mix-solutions for six different pairs of the enzymes consisting of: protease (2.5%): cellulase (2.5%), protease (2.5%): viscozyme (2.5%), protease (2.5%): α-amylase (2.5%), cellulase (2.5%): viscozyme (2.5%), cellulase (2.5%): α-amylase (2.5%), and viscozyme (2.5%): α-amylase (2.5%). The solutions were prepared, processed, and stored as mentioned in the previous step.

#### 2.5.3 Chia seeds pretreatment with enzymes models

To determine the effect of individual enzyme at different concentrations, 200 mg of the chia seeds were placed in 10 mL of the optimal solvent system of AC: H_2_O (70:30) mixture, added with 5 mL from respective enzyme, and placed in an oven at 40°C for 4hr. For a comparative analysis and to rule out the effect of water on phenolic extraction, a parallel study was performed where the individual aqueous enzymes were added (15 mL) directly to the chia seeds samples (200 mg). For validation of both models, a blank was added for optimal solvent system of AC: H_2_O (chia seeds sample in 70:30 acetone and water without enzyme) and water extraction model (chia seeds sample in water without enzymes). A total of 14-samples were prepared and following the pre-treatment of the samples for the predetermined time, all the samples were UA extracted employing the previously determined parameters for extraction.

For the mix-enzyme pairs, the chia seeds (200 mg) were added with 15 mL of the solvents. Six samples were prepared for the mix-enzymes models (MEM): I [5 mL AC: H_2_O + 5 mL protease (2.5%) + 5 mL cellulase (2.5%)], II [5 mL AC: H_2_O + 5 mL protease (2.5%) + 5 mL viscozyme (2.5%)], III [5 mL AC: H_2_O + 5 mL Protease (2.5%) + 5 mL amylase (2.5%)], IV [5 mL AC: H_2_O + 5 mL cellulase (2.5%) + 5 mL viscozyme (2.5%)], V [5 mL AC: H_2_O + 5 mL cellulase (2.5%) + 5 mL amylase (2.5%)], and VI [5 mL AC: H_2_O + 5 mL viscozyme (2.5%) + 5 mL amylase (2.5%)]. A blank consisting of 200 mg chia seeds sample in 15 mL of water was prepared to observe the comparative effect. The samples were processed for pre-treatment and extracted alike previous step. All the experiments were performed in triplicate (*n = 3*) and results are presented as mean ± standard deviation

All the samples after UA-extraction were filtered (20 mm cellulose filters), centrifuged (13000 rpm for 15 min), and the top layer was diluted at required concertation. The diluted solutions were syringe-filtered (2μ) and subjected to UPLC analysis for phenolic determination.

### 2.6 Statistical models

The statistical models of descriptive analysis along with paired sample *t*-test and K-mean cluster analysis were used to determine the significant differences for the effect of enzymes on the phytochemical composition of chia seeds. The statistical package for the social sciences (SPSS v 27.0) was utilized to compute the data.

## 3 Results

### 3.1 UA-MDMV for chia phenolic

#### 3.1.1 UA-MD

The UA-MD revealed more amount (ppm) for RA (41.30), FA (2.58), cGA (20.85), and KF (0.04) in AC: H_2_O (70:30) whereas, QT (0.37 ppm) was seen more in EtOH (100%) solvent. For an individual phenolic yield, the ascending order was (ppm): RA (288.97) > cGA (106.97) > FA (8.70) > QT (1.10) > KF (0.07).

The yield for phenolic in an individual solvent, exhibited an amount of 65.08 ppm for the AC: H_2_O (70:30) followed by an amount of 54.01 ppm in the AC: H_2_O (50:50) system. For a cumulative yield, more amount (168.41 ppm) of phenolic (cGA, RA, FA, QT, KF) was observed in the AC: H_2_O mixture (70:30). The data for UA-MD is shown in detail in Table 1.

**Table 1:** UA-MD parameters with solvent and its combinations used for phenolic extraction; the data indicated herein used triplicate samples (*n = 3*) with mean ± SD.

**S#**	**Solvent/s**	**Composition**	**Sample amount**	**Solvent volume**	**US parameters**	**Phenolic (ppm)**					**Phenolic yield/ solvent (ppm)**	**Phenolic yield/solvent mixture (ppm)**
						**cGA**	**RA**	**FA**	**QT**	**KF**		
1	AC	100%	100 mg	10 mL	amplitude (40%); pulse (10 s:5s); time (5 min)	0.32	14.05	0.00	0.00	0.00	*14.37*	*NA*
2	EtOH					0.00	13.27	1.28	0.37	0.00	*14.92*	
3	H_2_O					14.81	25.65	1.99	0.00	0.00	*42.44*	
4	AC: EtOH	50:50%				0.00	12.61	0.75	0.00	0.00	*13.35*	*40.49*
5		30:70%				0.00	12.53	0.95	0.00	0.00	*13.48*	
6		70:30%				0.00	12.77	0.89	0.00	0.00	*13.66*	
7	AC: H_2_O	50:50%				15.66	38.18	0.07	0.08	0.02	*54.01*	*168.41*
8		30:70%				17.31	31.87	0.05	0.07	0.02	*49.32*	
9		70:30%				20.85	41.30	2.58	0.31	0.04	*65.08*	
10	EtOH: H_2_O	50:50%				13.50	29.61	0.09	0.08	0.00	*43.29*	*125.18*
11		30:70%				14.99	25.54	0.06	0.10	0.00	*40.68*	
12		70:30%				9.54	31.58	0.00	0.09	0.00	*41.21*	
*Total yield for individual phenolic (ppm)*						*106.97*	*288.97*	*8.70*	*1.10*	*0.07*	*405.81*	

#### 3.1.2 UA-MV

The chia seeds from USA origin revealed more yield for the extract (0.35 g) as well as the phenolic of RA (46.96 pm), cGA (28.95 ppm), and QT (0.58 ppm). FA was seen more in Saudi Arabia-origin (26.96 ppm) whereas, KF was observed more in Ecuador-origin (0.54 ppm) chia seeds samples. The total yield (ppm) for individual phenolic in chia seeds samples (*N = 11*) revealed an ascending order of: RA (385.77) > cGA (86.33) > FA (40.51) > KF (4.13) > QT (1.70). The phenolic yield/origin exhibited a cumulative amount (ppm) of: USA (78.67) > Saudi Arabia (69.18) > Ecuador (52.49) > India (50.59)> (remaining < 50 ppm). The data for UA-MV is shown in detail in Table 2.

**Table 2:** UA-MV for the developed extraction method with extraction of the phenolic compounds from different origin chia seeds; data generated in the table represents triplicate samples (*n = 3*) with mean ± SD.

**S#**	**Code**	**Origin**		**Solvent volume**	**Amount**		**US parameters**	**Extract yield (g)**	**Phenolic (ppm)**							**Phenolic yield/origin (ppm)**
									**cGA**	**RA**		**FA**	**QT**	**KF**		
1	C1	Saudi Arabia		AC:H2O (70:30); 60 mL	10 g		amplitude (40%); pulse (10 s:5s); time (5 min)	0.27	6.99	34.90		26.96	0.03	0.29		*69.18*
2	C2	Ecuador						0.26	7.15	42.70		2.06	0.03	0.54		*52.49*
3	C3	Bahrain						0.17	3.74	27.23		0.81	0.11	0.35		*32.24*
4	C4	India						0.24	7.47	40.39		2.25	0.23	0.25		*50.59*
5	C5	Argentina						0.24	6.14	34.06		1.33	0.16	0.35		*42.04*
6	C6	Mexico						0.25	8.88	36.78		1.93	0.19	0.40		*48.18*
7	C7	Peru (yellowish)						0.24	5.65	34.33		0.09	0.13	0.28		*40.49*
8	C8	Peru (brownish)						0.23	6.49	35.83		1.99	0.14	0.37		*44.80*
9	C9	Bolivia						0.24	0.00	18.62		0.05	0.08	0.53		*19.29*
10	C10	USA						0.35	8.95	46.96		1.79	0.58	0.40		*58.67*
11	C11	Spain						0.23	4.87	33.97		1.25	0.04	0.37		*40.49*
*Total yield for individual phenolic (ppm)*									*66.33*	*385.77*		*40.51*	*1.70*	*4.13*		*627.59*

**Descriptive statistics**																
**Variable**			**Minimum**			**Maximum**		**Sum**			**Mean**				**Standard deviation**	

**Extract yield**			0.17			0.35		2.72			0.28				0.04	
**cGA**			0.00			28.95		86.33			7.85				7.38	
**RA**			18.62			46.96		385.77			35.07				7.52	
**FA**			0.05			26.96		40.51			3.68				7.76	
**QT**			0.03			0.58		1.72			0.16				0.15	
**KF**			0.25			0.54		4.13			0.37				0.09	

### 3.2 UPLC for chia phenolic

#### 3.2.1 Optimization of UPLC analytical method

**Fig. 1:**
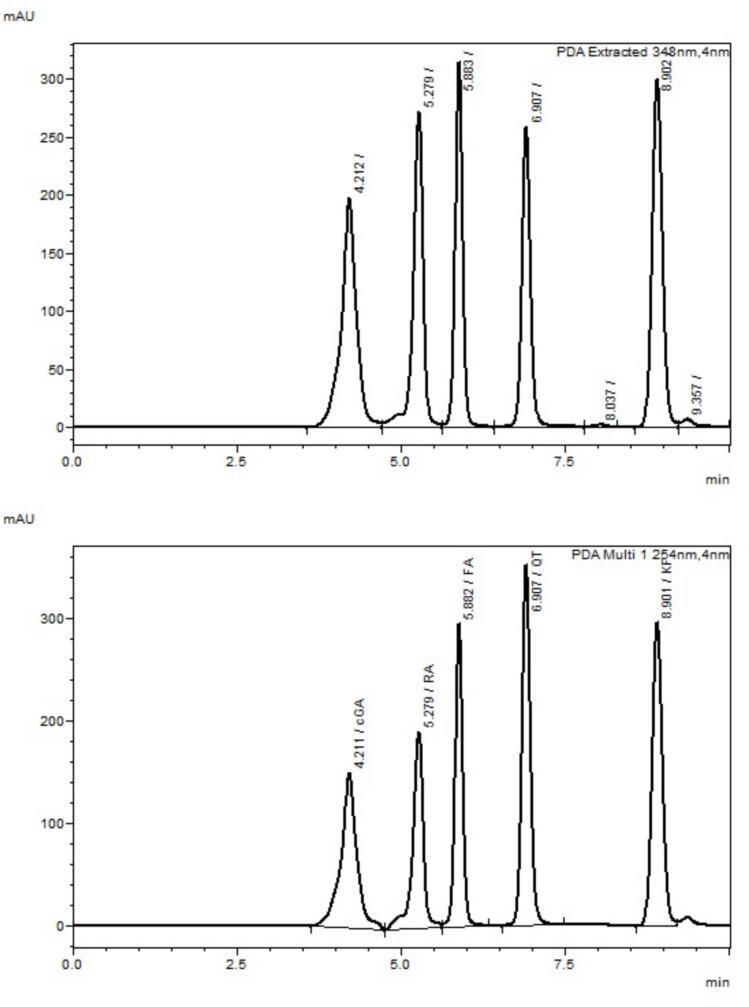
Representative chromatogram with rt for the simultaneous determination of chia seeds phenolic (cga, ra, fa, qt, and kf) at 348 nm and 245 nm wavelength.

**Fig. 2:**
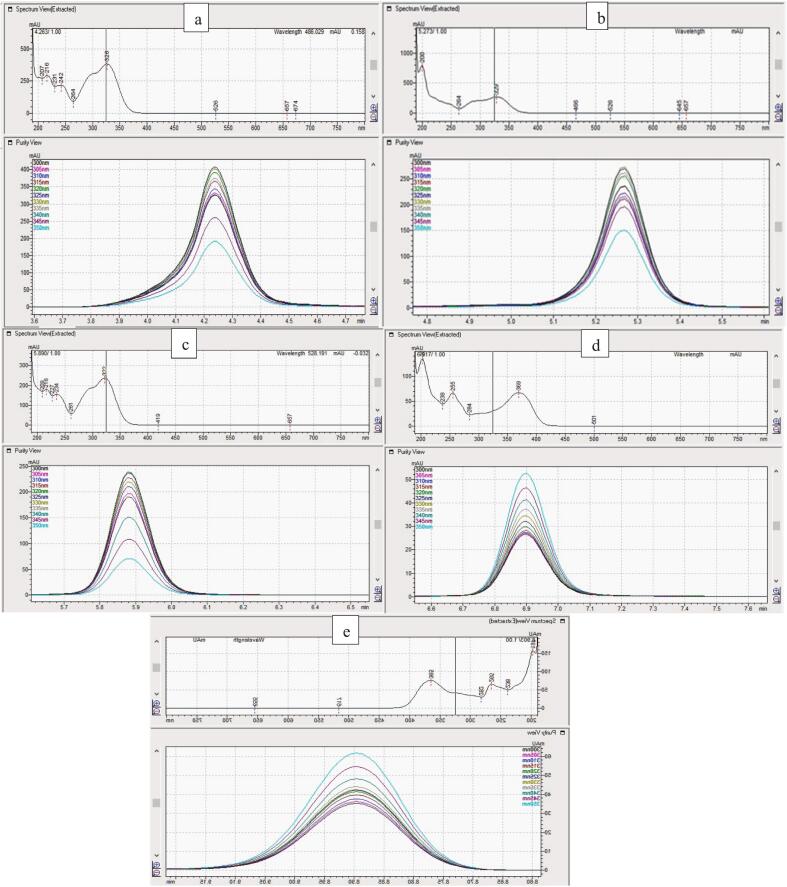
UV-profiles with different absorption regions for cGA (a), RA (b), FA (c), QT (d), and KF (e).

The chromatographic method for the simultaneous determination of the phenolic finalized the conditions for separation: column [Restek 150mmX4.6 mm, 3 μ m], mobile phase [B (50% of 0.1% formic acid in ACN) and A (50% of 0.1% formic acid in H_2_O)], elution (gradient), runtime (10 min), injection volume (5 μ L), and column oven temperature (35°C). The retention time (Rt) observed was 4.21 (cGA), 5.28 (RA), 5.88 (FA), 6.91 (QT), and 8.90 (KF) whereas, the peak resolution observed were (Rs) 0.00 (cGA), 3.22 (RA), 2.43 (FA), 4.35 (QT), and 7.16 (KF). The tailing factor was seen in the range of 0.72–1.09. The wave lengths for the UV detector were set in the range of 190–800 nm whereas, the extracted chromatograms at 348 nm and 254 nm are shown in Fig. 1. The absorption for phenolic at different wavelengths are extracted and shown in Fig. 2 **(a-e)**. The data regarding the UPLC method development is provided in Table 3.

**Table 3:** UPLC-MDMV for the analytical method to simultaneously quantify the phenolic compounds in chia seed samples; data presented is mean ± SD (*n = 3*).

**Parameters**	**Phenolic**				
	**cGA**	**RA**	**FA**	**QT**	**KF**
**Mobile phase**	A (50% H2O 0.1%FA) and B (50% ACN 0.1%FA)				
**Flow rate**	0.3 mL/min				
**Injection volume**	5 μ L				
**Column oven temperature**	35°C				
**Column dimensions**	Restek [150 mm X 4.6 mm, 3 μ m]				
**Retention time (Rt)**	4.21	5.28	5.88	6.91	8.90
**λ-nm**	253 & 348 (UV range 190–600 nm)				
**Resolution**	0.00	3.22	2.43	4.35	7.16
**Tailing factor**	0.98	0.72	1.03	1.07	1.09
**Accuracy (%)**	95–106%				
**Rel. SD (%)**	1.68–8.97				
**Linearity (ppm)**	5–200		1–100		
**r2**	0.97	0.99	0.97	0.99	0.99
**LOD (ppm)**	0.1–0.14	0.09–0.14	0.03–0.06	0.02–0.04	0.03–0.04
**LOQ (ppm)**	0.29–0.42	0.26–0.43	0.1–0.17	0.06–0.11	0.08–0.13
**Regression Equation**	Y = 25,529.0X + 0	Y = 19,676.1X + 0	Y = 36,570.9X + 0	Y = 66,201.7X + 0	Y = 62,877.7X + 0

#### 3.2.2 Validation of UPLC analytical method

**Fig. 3:**
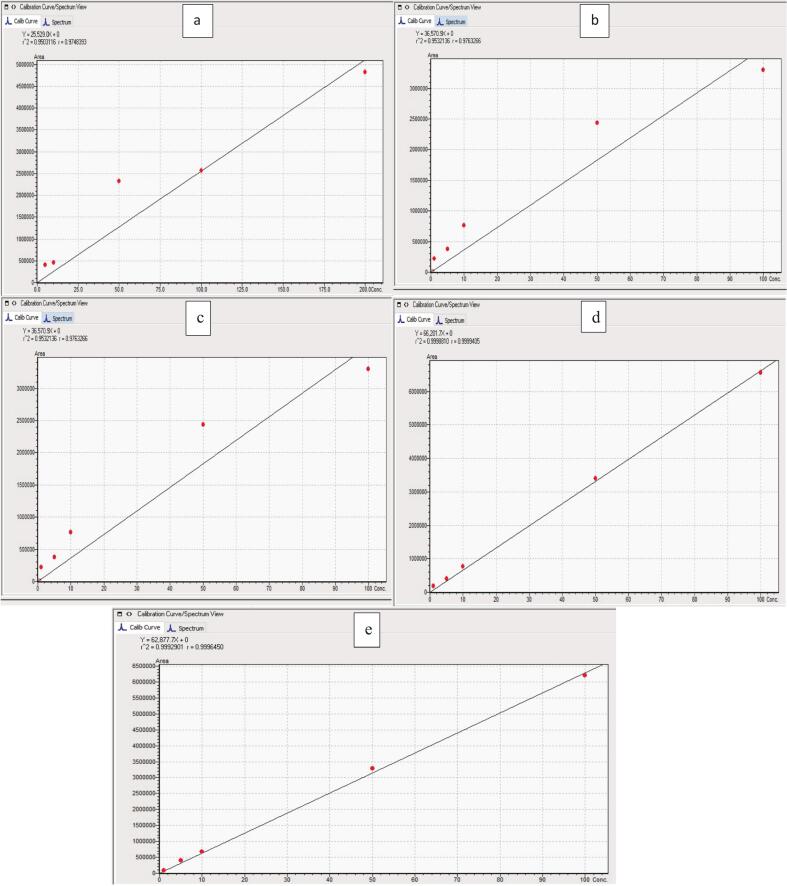
R^2^-values with regression equations for cGA (a), RA (b), FA (c), QT (d), and KF (e).

The method was validated with accuracies in the range of 95–106%, r^2^-values (0.97–0.99), LOD (0.02–0.14 ppm), LOQ (0.06–0.43), and relative standard deviations (Rel. SD%) in the range of 1.68–8.97. The data regarding the UPLC phenolic method validation for chia seeds consisting of the regression equation for individual phenolic is shown in Table 3 whereas, the figures for the determination of coefficients are shown in Fig. 3.

### 3.3 Phenolic extraction using individual enzyme models (EM)

#### 3.3.1 General yields for phenolic in individual EM

The phenolic yield for water-based EM showed more amount of cGA (2.71 ppm) in 5%cellulase, RA (6.86 ppm) in 5% α-amylase, and FA (0.51 ppm) in 2.5% α-amylase enzyme. No QT and KF were detected in the water-based EM. The total yield for individual phenolic constructed an order: RA (44.81 ppm) > cGA (9.57 ppm) > FA (3.74 ppm) whereas, the phenolic yield in individual enzyme exhibited the order: α-amylase (18.30 ppm) > viscozyme (13.92 ppm) > cellulase (13.76 ppm) > protease (12.14 ppm). The blank sample for water-based EM (C0) resulted a lower amount for cGA (1.64 ppm), RA (1.62 ppm), and FA (0.26 ppm). The data for the water-based EM for phenolic is shown in Table 4.

**Table 4:** Extraction of chia seed phenolic using the individualenzymes (EM) model in water and optimal solvent systems of AC: H_2_O; data was recorded in triplicate (*n = 3*) and presented as mean ± SD.

**S#**	**Enzymes**	**Code**	**Conc.**	**Sample and solvent**	**Pre-treatment conditions**	**US parameters**	**Phenolic (ppm)**					**Phenolic yield/ enzyme (ppm)**	
							**cGA**	**RA**	**FA**	**QT**	**KF**		
**Extraction using enzymes in water solvent**													
1	Protease	Pw1	1%	200 mg in 15 mL (10 mL water + 5 mL enzyme solution)	40C for 4 h followed by enzyme inhibition	amplitude (40%); pulse (10 s:5s); time (3 min)	0.00	14.77	1.19	0.00	0.00	*15.95*	*36.43*
2		Pw2	2.5%				0.00	15.25	1.02	0.00	0.00	*16.27*	
3		Pw3	5%				0.27	2.74	1.19	0.00	0.00	*4.20*	
4	Cellulase	Cw1	1%				2.97	7.79	0.50	0.00	0.00	*11.25*	*41.29*
5		Cw2	2.5%				4.87	7.40	0.65	0.00	0.00	*12.91*	
6		Cw3	5%				8.13	8.46	0.54	0.00	0.00	*17.12*	
7	Viscozyme	Vw1	1%				0.95	14.97	0.00	0.00	0.00	*15.92*	*41.76*
8		Vw2	2.5%				2.53	14.26	1.04	0.00	0.00	*17.83*	
9		Vw3	5%				4.50	2.53	0.98	0.00	0.00	*8.00*	
10	Amylase	Aw1	1%				1.85	6.63	1.22	0.00	0.00	*9.70*	*54.89*
11		Aw2	2.5%				1.94	19.06	1.54	0.00	0.00	*22.54*	
12		Aw3	5%				0.70	20.57	1.38	0.00	0.00	*22.65*	
13	C0	Without enzyme					1.64	1.62	0.26	0.00	0.00	*3.52*	*61.66*
*Total yield for individual phenolic in aqueous enzymes*							*28.71*	*134.42*	*11.23*	*0.00*	*0.00*	*177.88*	
**Extraction using enzymes in AC: H_2_O (70:30) solvent**													
1	Protease	Ps1	1%	200 mg in 15 mL (10 mL AC:H_2_O + 5 mL enzyme solution)	40C for 4 h followed by enzyme inhibition	amplitude (40%); pulse (10 s:5s); time (3 min)	19.66	2.10	1418.36	0.23	0.16	*1440.50*	*1472.84*
2		Ps2	2.5%				10.45	2.23	0.08	0.00	0.06	*12.81*	
3		Ps3	5%				16.97	2.43	0.10	0.00	0.02	*19.52*	
4	Cellulase	Cs1	1%				20.40	2.30	0.10	0.00	0.03	*22.83*	*59.43*
5		Cs2	2.5%				22.40	2.90	0.85	0.00	0.09	*26.23*	
6		Cs3	5%				7.05	3.17	0.06	0.00	0.08	*10.37*	
7	Viscozyme	Vs1	1%				15.56	2.13	1566.71	0.00	0.00	*1584.39*	*1672.49*
8		Vs2	2.5%				19.63	23.91	0.69	0.00	0.04	*44.27*	
9		Vs3	5%				21.66	22.05	0.06	0.00	0.05	*43.83*	
10	Amylase	As1	1%				15.49	2.41	1344.80	0.00	0.02	*1362.71*	*1437.89*
11		As2	2.5%				17.03	2.10	0.10	0.00	0.02	*19.25*	
12		As3	5%				33.53	21.62	0.78	0.00	0.00	*55.93*	
13	CS0	Without enzyme					25.44	8.48	6.27	0.76	0.05	*15.63*	
*Total yield for individual phenolic in optimal solvent system enzymes*							*186.27*	*89.36*	*4332.68*	*0.23*	*0.58*	*4642.65*	

#### 3.3.2 Water-based EM

##### 3.3.2.1 EM for protease

The protease resulted more yield for RA (5.08 ppm) at 2.5% followed by FA (0.40 ppm) at 1% and 5%. The yield for cGA was 0.09 ppm at 5%. The phenolic yield (ppm) in individual enzyme concentration (%) revealed: 5.42 (2.5%) > 5.32 (1%) > 1.40 (5%).

##### 3.3.2.2 EM for cellulase

The cellulase enzyme exhibited more yield for RA (2.82 ppm) and cGA (2.71 ppm) at 5% concentration followed by FA (0.22 ppm) at 2.5%. The yield for phenolic (ppm) in individual enzyme concentration (%) was note with order of: 5.71 (5%) > 4.30 (2.5%) > 3.75 (1%).

##### 3.3.2.3 EM for viscozyme

For viscozyme more RA (4.99 ppm) was observed at 1% followed by cGA (1.50 ppm) at 5% and FA (0.35 ppm) at 2.5% concentration. The order for phenolic yield (ppm) in individual enzyme (%) used was: 5.94 (2.5%) > 5.31 (1%) > 2.67 (5%).

##### 3.3.2.4 EM for α-amylase

The yield for phenolic (ppm) in cellulase enzyme revealed more amount for RA (6.86) at 5%, cGA (0.65) at 2.5%, and FA (0.51) at 2.5% concentration. The total yield for phenolic (ppm) in individual enzyme (%concentration) suggested the order: 7.55 (5%) > 7.51 (2.5%) > 3.23 (1%).

#### 3.3.3 AC: H_2_O-based EM

##### 3.3.3.1 General yields for phenolic

The yields for phenolic in AC: H_2_O-based EM showed more amount for FA (1566.71 ppm) in 1% viscozyme, cGA (33.53 ppm) in 5% α-amylase, RA (23.91 ppm) in 2.5% viscozyme, QT (0.23 ppm) in 1% protease, and KF (0.16 ppm) in 1%protease. The order for total yield of individual phenolic (ppm) was: FA (4332.68) > cGA (186.27) > RA (89.36) > KF (0.58) > QT (0.23). The yield for phenolic (ppm) in individual enzyme suggested the order: viscozyme (1672.49) > protease (1472.84) > α-amylase (1437.89) > cellulase (59.43). The blank sample for AC: H_2_O-based EM resulted 8.48 ppm (cGA), 6.27 ppm (RA), 0.76 ppm (FA), 0.06 ppm (KF), and 0.05 ppm (QT), as shown in Table 4.

##### 3.3.3.2 EM for protease

The phenolic yield for protease was seen higher at 1% for FA (1418.36 ppm), cGA (19.66 ppm), QT (0.23 ppm), and KF (0.16 ppm) whereas, for RA more yield (2.43 ppm) was observed at 5% concentration. The total yield for the phenolic (ppm) in protease enzyme (%concentration) showed the order: 1% (1440.50) > 5% (19.52) > 2.5% (12.81).

##### 3.3.3.3 EM for cellulase

The cellulase enzyme resulted more yield for phenolic (ppm) at 2.5% for cGA (22.40), FA (0.85), and KF (0.09). More amount for RA (3.17 ppm) was observed at 5% whereas, no QT was seen at any of the concentrations. The order for the total phenolic yield (ppm) in individual enzyme (%concentration) was: 2.5% (26.23) > 5% (22.83) > 1% (10.37).

##### 3.3.3.4 EM for viscozyme

For viscozyme, more yield (ppm) resulted in 1% for FA (1566.71), 2.5% for RA (23.91) and, 5% for cGA (21.66) and KF (0.05). QT was not observed for any of the concentrations used. The total yield for phenolic (ppm) in individual enzyme (%concentration) resulted the order: 1% (1584.39) > 2.5% (44.27) > 5% (43.83).

##### 3.3.3.5 EM for α-amylase

The concentration for α-amylase resulting more yield was 1% for FA (1344.80 ppm) and KF (0.02 ppm) and, 5% for cGA (33.53 ppm) and RA (21.62 ppm). The total yield phenolic yield (ppm) in an individual enzyme (%concentration) exhibited the order: 1% (1362.71) > 5% (55.93) > 2.5% (19.25).

### 3.4 Water-based vs AC: H_2_O based EM comparison

The general yields for water-based EM resulted more cGA and RA in 5% α-amylase enzyme and FA in 2.5% α-amylase enzyme whereas, AC: H_2_O-based EM revealed more amount for cGA in 2.5% α-amylase enzyme, RA and FA in 1 and 2.5% viscozyme, respectively. Water-based EM showed no QT and KF extraction whereas, the later model extracted more QT and KF in 1%protease.

On an individual basis, the phenolic of RA and cGA were higher for water-based whereas, FA and cGA were higher for AC: H_2_O-based EM.

The phenolic yield in an individual enzyme was seen more for α-amylase followed by viscozyme in water-based EM whereas, for AC: H_2_O more yield for phenolic was observed in viscozyme followed by α-amylase enzyme.

### 3.5 Mix-enzymes models (MEM)

#### 3.5.1 General yields

The MEM revealed more yield (ppm) for total phenolic amount on individual basis to be: FA (2404.67) > RA (118.56) > cGA (35.04) > KF (0.62) > QT (0.23). The highest yield for individual phenolic exhibited more amount (ppm) for FA (504.31) followed by RA (29.57), cGA (14.15), KF (0.29), and QT (0.08).

The order for phenolic with high amount on an individual basis in the MEM was: FA (MEM-III) > RA (MEM-IV) > cGA (MEM-VI) > KF (MEM-V) > QT (MEM-III). The data for MEM (I-VI) is provided in Table 5.

**Table 5:** Extraction of chia seed phenolic using themix-enzymes (MEM) model in water and optimal solvent systems of AC: H_2_O; data provided in table is (*n = 3*) with mean ± SD.

**S#**	**Enzymes combination**	**Code**	**Enzymes mixture in solvent system (AC:H2O; 70:30)**	**Sample and solvent**	**Pre-treatment conditions**	**US parameters**	**Phenolic (ppm)**					**Phenolic yield/ enzyme (ppm)**
							**cGA**	**RA**	**FA**	**QT**	**KF**	
1	Protease+ cellulase	MEM1	5 mL protease (2.5%) + 5 mL cellulase (2.5%) + 5 mL solvent system	100 mg in 15 mL enzymes + solvents mixture	40C for 4 h followed by enzyme inhibition	amplitude (40%); pulse (10 s:5s); time (3 min)	0.00	34.88	353.72	0.00	0.00	*388.60*
2	Protease+ viscozyme	MEM2	5 mL protease (2.5%) + 5 mL viscozyme (2.5%) + 5 mL solvent system				9.39	20.80	360.87	0.04	0.04	*391.13*
3	Protease+ amylase	MEM3	5 mL protease (2.5%) + 5 mL amylase (2.5%) + 5 mL solvent system				11.51	11.22	504.31	0.08	0.03	*527.14*
4	Cellulase+ viscozyme	MEM4	5 mL cellulase (2.5%) + 5 mL viscozyme (2.5%) + 5 mL solvent system				0.00	29.57	464.23	0.00	0.02	*493.83*
5	Cellulase+ amylase	MEM5	5 mL cellulase (2.5%) + 5 mL amylase (2.5%) + 5 mL solvent system				0.00	15.99	344.12	0.08	0.29	*360.48*
6	Viscozyme+ amylase	MEM6	5 mL viscozyme (2.5%) + 5 mL amylase (2.5%) + 5 mL solvent system				14.15	6.11	377.42	0.02	0.24	*397.93*
7	No enzyme	MEM0	10 mL d·H_2_O + 5 mL solvent system				7.64	3.45	90.00	0.00	0.00	*2559.11*
*Total yield for individual phenolic in optimal solvent system enzymes*							*35.04*	*118.56*	*2404.67*	*0.23*	*0.62*	

#### 3.5.2 MEM-I

For MEM-I, FA (353.72) followed by RA (34.88). The phenolic of cGA, QT, and KF were not extracted seen in MEM-I model. The total yield for phenolic/enzyme showed 388.60 ppm for MEM-I.

#### 3.5.3 MEM-II

The yields for MEM-II resulted more amount for FA (360.87 ppm), followed by RA (30.80 ppm), cGA (9.39 ppm), QT (0.04 ppm), and KF (0.04 ppm). The total yield for phenolic/enzyme showed an amount of 391.13 for MEM-II.

#### 3.5.4 MEM-III

MEM-III exhibited more yield in the descending order of: FA (504.31 ppm) > RA (11.51 ppm) > cGA (11.22 ppm) > QT (0.08 ppm) > KF (0.03 ppm). The total phenolic yield for MEM-III observed was 527.14 ppm.

#### 3.5.5 MEM-IV

The order for phenolic amount in MEM-IV constructed a descending order of: FA (464.23 ppm) > RA (29.57 ppm) > KF (0.02 ppm). The phenolic of cGA and QT was not observed for MEM-IV. The total yield for phenolic in MEM-IV was 493.83 ppm.

#### 3.5.6 MEM-V

The yield for phenolic in MEM-V exhibited the descending order of: FA (344.12 ppm) > RA (15.99 ppm) > KF (0.29 ppm) > QT (0.08 ppm). cGA was not seen for MEM-V samples whereas, the total yield for phenolic was 360.48 ppm.

#### 3.5.7 MEM-VI

The descending order for the phenolic yield in MEM-VI was: FA (377.42 ppm) > cGA (14.15 ppm) > RA (6.11 ppm) > KF (0.24 ppm) > QT (0.02 ppm). The total yield for phenolic/enzyme in MEM-VI resulted 397.93 ppm for the sample.

### 3.6 Statistical models results

#### 3.6.1 Descriptive statistics

**Fig. 4:**
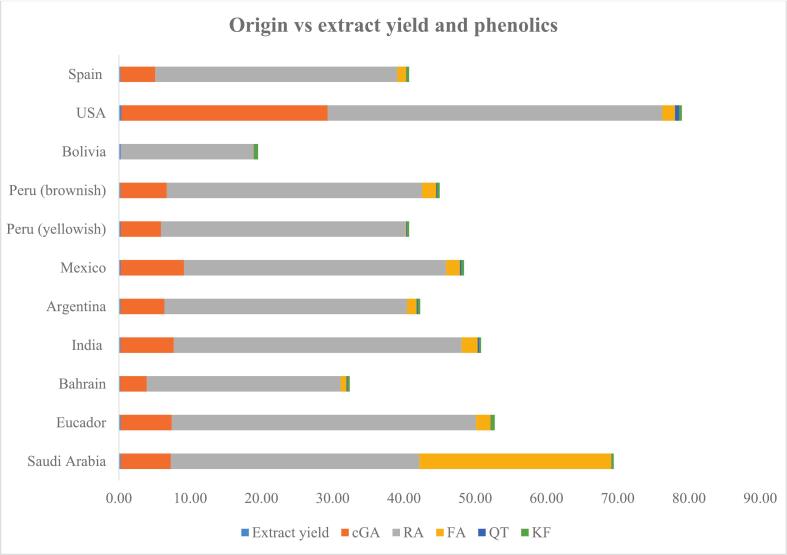
Comparative representation of the extract yield and phenolic compounds in different origin chia seeds samples.

The descriptive statistics for the twelve different geographical origin of chia seeds (UAMV) revealed a mean 0.28 g with sum(±SD) of 2.72 g (±0.04) in the range (minimum–maximum) of 0.17–0.35 g for the extract yield. The sum and mean(±SD) for the phenolic was (ppm): cGA [86.33 and 7.85 (±7.38)]. RA [385.77 and 35.07 (±7.52)], FA [40.51 and 3.68 (±7.76)], QT [1.72 and 0.16 (±0.15)], and KF [4.13 and 0.37 (±0.09)]. The ranges for the phenolic (ppm) was (minimum–maximum): cGA (0.00–28.95), RA (18.62–46.96), FA (0.05–26.96), QT (0.03–0.58), and KF (0.25–0.54). The data for descriptive statistics is shown in Table 2 whereas, the data for the 12-origins chia seeds samples is presented in Fig. 4.

#### 3.6.2 Paired sample *t*-test

The paired sample *t*-test was used to find the differences for the type of enzyme vs the phenolic yield in chia seeds. All the six pairs exhibited significant differences for the change in enzyme vs the phenolic yield: enzyme – cGA (*M = 6.08*; CI of 2.44–9.66, at *P =* 0.002), enzyme – RA (*M = 4.09*; CI of 0.016–8.16, at *P =* 0.049), enzyme – FA (*M = -209.45*; CI of −374.51–44.40, at *P =* 0.015), enzyme – QT (*M =* 15.48; CI of 12.20–18.77, at *P=<*0.001), and enzyme – KF (*M =* 15.46; CI of 12.19–18.73, at *P=<*0.001). The detailed data for paired sample *t*-test is shown in Table 6.

**Table 6:** Results for the paired sample*t*-test and K-mean cluster analysis of the chia seed samples; * negative mean difference found in the paired *t*-test indicates that phenolic concentrations of enzymatically treated samples were higher compared to the control samples.

**Paired sample *t*-test**							
**Variables**	**Mean**	**Std. dev**	**95% CI**			***t***	***P***
			**Lower**		**Upper**		
Pair 1: Enzyme − cGA	6.08	9.67	2.44		9.66	3.42	0.002
Pair 2: Enzyme − RA	4.09	10.90	0.016		8.16	2.05	0.049
Pair 3: Enzyme − FA	−209.45*	442.01	−374.51		−44.40	−2.59	0.015
Pair 4: Enzyme − QT	15.48	8.79	12.20		18.77	9.64	<0.001
Pair 5: Enzyme − KF	15.46	8.77	12.19		18.73	9.66	<0.001

**K-mean cluster analysis**							
**Variables**	**F-value**	**Significance**		**Clusters**		**Samples**	

Z score: cGA	7.240	<0.001		1		2	
Z score: RA	8.126	<0.001		2		5	
Z score: FA	55.834	<0.001		3		16	
Z score: QT	27.420	<0.001		4		2	
Z score: KF	39.864	<0.001		5		1	
				6		4	
				Total		30	

#### 3.6.3 K-mean cluster analysis

**Fig. 5:**
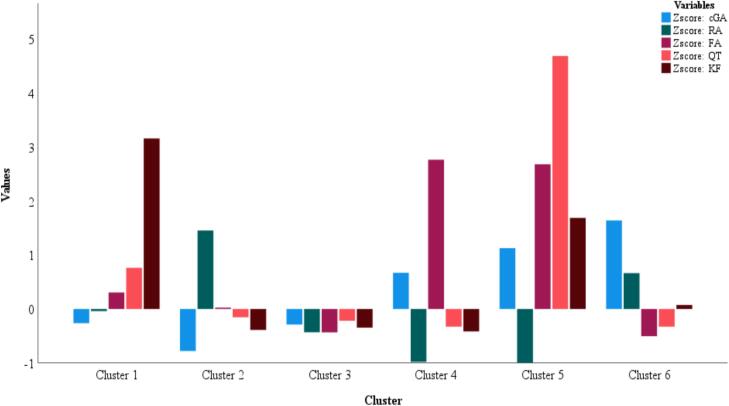
Distribution of the phenolic compounds in chia seeds samples into various clusters.

To perform the K-means cluster analysis, the SPSS program (V 27.0) was used. Prior to run the test, all the variables of cGA, RA, FA, QT, and KF were standardized using the Z-score method for equal weighting. The default Euclidean distance method was used for similarity measurement. The analysis was conducted using six-clusters based on the distribution of the phenolic variables. The program was set to allow for a maximum of 10 iterations and random start was chosen for the cluster centers. The convergence criterion was set to automatically stop the iterations when the maximum absolute change in the cluster centers was less than 0.000. Herein, this was achieved after the third iteration whereas the minimum distance between initial centers was 3.535. The K-mean analysis categorized the samples into six different clusters: cluster 1 (2), cluster 2 (5), cluster 3 (16), cluster 4 (2), cluster 5 (1), and cluster 6 (4). The sample with more phenolic (cGA, FA, highest amount of QT, and KF) was placed in cluster 5 (Ps1). This shows more phenolic in protease (1%) based solvent system developed for extraction. Only two samples (MEM5 and MEM6) were placed in cluster 1, showing the highest amount for KF. These two represents the solvents system containing a mixture of celullase + amylase and viscozyme + amylase, respectively. For RA, the highest amount was seen in cluster 2 consisting of five samples of Aw2, Aw3, MEM1, MEM2, and MEM4 representing the amylase enzyme at concentrations of 2.5 and 5% in addition to the mix enzyme systems of protease, cellulase, and viscozyme. Cluster 4 exhibited 2-samples with more amount of FA (Vs1 and As1). This suggest the presence of more amount of FA in 1% viscozyme and 1% amylase enzymes in the solvent systems. Likewise, the samples with more cGA amount were placed in cluster 6 consisting of 4-samples of Cs2, Vs2, Vs3, and As3. The remaining 16-samples were placed in cluster 3, showing either a lack of very low amount of the tested phenolic. The details regarding the enzymes vs samples clustering is shown in Table 6 and Fig. 5.

## 4 Discussion

Current study comprehensively evaluated the effect of ultrasound dismembrartor-based extraction (UA-MD and UA-MV) with enzymes-assisted (individual and mixed-enzymes) models on the release as well as quantification of the key phenolic of: chlorogenic acid (cGA), rosmarinic acid (RA), ferulic acid (FA), quercetin (QT), and kaempferol (KF) in chia seeds. The findings herein represent substantial differences in terms of phenolic yield in chia seeds, suggested due to the usage of unique extraction model, various solvents compositions, and enzymes models.

For the release and extraction of phenolic compounds form the chia seeds matrix, ultrasound dismembrator with green solvents of acetone (AC), ethanol (EtOH), and water (H_2_O), and its various combinations were employed. UA is well-known for an enhanced yield in the field of phytochemical extraction where it works through cell disruption of the matrix, accelerating the mass transfer, and phenolic diffusion as observed previously for chia seeds [23]. The results for the UA-MD supported the decisive role for the polarity of the solvent in phenolic extraction and recovery. A high cumulative yield for phenolic was observed in the AC: H_2_O (70:30) system, outperforming more ethanol- or aqueous-based solvents systems. The comparatively enhanced yield for the moderately polar system of AC: H_2_O system align the principle of extraction science and previously reported chia seeds results where a significantly improved extractability for chia seeds phenolic was witnessed [12]. It is worthy to mention that the enhanced yield for phenolic compounds from chia seeds, observed in this study, is due to the optimal polarity of the AC: H_2_O mixture under the acoustic cavitation effects of UA. The acoustic cavitation is considered among the major mechanisms for UA responsible for the enhanced yields [21].

To determine the presence and quantify the amount of phenolic compounds in the UA-MD extracted samples, an in-house analytical method was developed for a simultaneous determination of the phenolic using UPLC-DAD. The UPLC method was developed and validated as per ICH guidelines. The method exhibited a short runtime of eight minutes for the simultaneous determination of the chai phenolic with LOD, LOQ, peak separation, and Rs in the defined permissible limits. This indicate the good accuracy, sensitivity, and precision for the developed method. Previous studies have reported the simultaneous determination for cGA, FA, and RA [19], and LCDAD-MSMS identification for phenolic compounds in chia seeds [4] however, none of the studies reported a simultaneous determination for cGA, RA, FA, QT, and KF in a single short runtime of 8 min. Herein, the chia phenolic are reported for the first time with a shorter runtime using the UA-MD followed by UA-MV in order to validate the developed method in chia seeds samples belonging to different geographical origins.

The UPLC-DAD quantification for the extracted samples (UA-MD) revealed the presence of all the phenolic compounds in these samples with a descending order of occurrence: RA > cGA > FA > QT > KF. The phenolic compounds quantified herein align the previous reports where the analytical investigations have identified the presence of chlorogenic acid, caffeic acid, ferulic acid, and rosmarinic acid along with quercetin and kaempferol in chia seeds [4], [6], [9], [12]. The successful quantification for chia phenolic indicates the validity for the developed UPLC-DAD method, confirming the characteristic phytochemical fingerprint for the chia seeds. As more cumulative yield for phenolic was seen in the AC: H_2_O system (70: 30), the extraction for chia seeds samples in UA-MV procedure used the same solvent. This approach help optimize the UA-MD based solvent system for chia seed phenolic extraction and to validate the UA method on a larger scale using the real world samples of chia seeds. In detail: chia seeds from eleven different geographical origins were extracted with the help of ultrasonic dismembrator using a high amount of the chia seeds samples and more volume of the AC: H2O system. The extracted samples subjected to UPLC-DAD analysis revealed the presence of all the tested phenolic compounds throughout the eleven different geographical origins chia seeds. Interestingly, a significant variation was observed for the concentration of individual phenolic across these chia seeds samples. In general, the yield for phenolic was observed in the descending order of: RA > cGA > FA > QT > KF whereas, the chia seeds sample from USA origin exhibited more extract yield and concentration for the phenolic of RA, cGA, and QT. On contrary, the phenolic of FA was seen more in Saudi Arabian-origin whereas, KF was present more in Ecuador-origin chia seeds. The cumulative yield for the phenolic compounds in respective origin of the chia seeds resulted a descending order of: US > Saudi Arabia > Ecuador > India. The outcomes indicate a huge variation for the amount of phenolic content in the chia seeds samples sourced from various geographical origins. Though the phytochemical variation in chia seeds prone to geographical origins is well documented [10], [24], it’s a first time study to report a comprehensive comparative evaluation for phenolic compounds in chia seeds from eleven different geographical samples. The unique environmental stresses to which chia seeds from the United States, Saudi Arabia, and Ecuador were exposed may have contributed to the varying degrees of enhancement of phenolic biosynthesis via well-characterized stress-responsive pathways, especially with regard to major phenolic acids. The phenolic biosynthesis is catalyzed by the shikimate-phenylpropanoid biosynthetic pathway, with critical regulatory steps catalyzed by enzymes such as phenylalanine ammonia-lyase, cinnamate 4-hydroxylase, and 4-coumarate, CoA ligase, all of which are transcriptionally activated by abiotic stresses to increase carbon flux through this pathway to produce phenolic. An increased exposure to ultraviolet radiation would be experienced by seeds from the United States and Ecuador at higher latitudes and from Ecuador at higher elevations which may activate specific UV-B photoreceptors such as: UVR8 to induce transcription of phenylpropanoid biosynthetic genes and produce flavonoids and hydroxycinnamates. This may be reinforced by UV-induced ROS to activate antioxidant defenses that produce phenolic antioxidants. More diurnal temperature variation, characteristic for some US growing regions, results in fluctuating thermal stresses that disrupt photosynthetic homeostasis and stimulate phenylpropanoid enzyme activities synergistically with light to increase phenolic compound synthesis. In contrast, seeds from Saudi Arabia experienced constant high heat and arid stresses that stimulate cellular ROS and abscisic acid-mediated drought signaling pathways, resulting in an increased phenylalanine ammonia-lyase and phenylpropanoid gene expression thus, increasing phenolic compound biosynthesis. In summary, these abiotic stresses of high UV radiation, extreme diurnal temperature fluctuation, heat, and drought converge on common signaling pathways that increase phenolic compound biosynthesis in all of these seeds via activation of the phenylpropanoid pathway. [4], [25], [26], [27], [28] The variation in geographical origin influences not only the micro- and macro-nutrients but the major phytochemicals in chia seeds due to numerous considerable factors of: change in soil composition and pH, altitude, climate, temperature, humidity, fertilizer used, storage, and shipment conditions etc. [29], [30]. Therefore, the variation in phenolic yields for different origins highlights the need for proper standardization of the chia seeds when collected from different places. The impact of environmental and post-harvest conditions on phenolic composition is well documented. For instance, the effect of climatic conditions, genotype, temperature, irrigation status, and storage conditions leading to variation in phenolic composition is reported previously in detail. [25], [31], [32], [33], [34], [35] The environmental influences such as temperature, solar irradiance, soil type, and water availability are known to influence the phenylpropanoid biosynthesis pathway which in turn influences phenolic compound biosynthesis. For example, abiotic stressors like reduced irrigation schedules and environmental extremes could trigger oxidative stress in plants, which in turn upregulates critical enzymes in phenolic compound biosynthesis such as phenylalanine ammonia-lyase to produce antioxidant phenolic compounds. The genotype-specific metabolic potential and environmental interactions could influence specific phenolic compound accumulation in chia seeds, leading to variations in antioxidant potential and phenolic composition of chia seeds from different geographical locations. [25], [31], [32], [33], [34], [35] Yet again, it is highly recommended to explore in-depth the agro- and environmental-factors responsible for the phenolic variation for chia seeds grown in Saudi Arabia in order to elucidate the effect on biosynthetic pathways for these phenolic in the seeds.

Further in this study, enzymes with different concentrations were employed to evaluate the effect of enzymes-assisted UA on the yield of chia phenolic. The enzymes were studied in two different models: individual (EM) and mix-enzymes models (MEM), to determine the comparative effect on phenolic yield. Briefly: manufacturer’s specifications were strictly adhered in terms of the optimal pH and temperature conditions for each enzyme in order to ensure the optimal enzymatic activity. Beside these fix parameters, the concentration of the enzymes (1, 2.5, and 5%) with extraction temperature and time as well as sample-to-enzyme ration were tested in-house, based on previous literature reports. Moreover, for each model of enzymes, a proper control was tested in parallel to rule out the effect of the solvent system on the phenolic content of the chia seeds. The EM data exhibited an enhanced liberation for the phenolic compounds, RA and FA in particular, compared to the control (solvent only). The water-based enzyme model showed a measurable increase for the cGA, RA, and FA with a lack of extraction for QT and KF. Whereas, the AC: H_2_O (70: 30) system revealed a dramatic increase for the phenolic compounds including QT and KF, specifically for FA in 1% viscozyme. The phenolic of cGA, FA, and RA were extracted more in the viscozyme and amylase enzyme system whereas, more amount of QT and KF were extracted in protease enzyme. The lack of QT and KF in water-based system is suggested due to the low polarity and sparing solubility in water [36]. This reveal the use of an appropriate enzyme to align the nature of the matrix for an enhanced release. For instance, the studies reported the use of glycosidase enzymes with more yield for the flavonoids of QT and KF showing the correct enzyme selection for enhancing the solubility and amount of mentioned flavonoids [37].

The finding is consistent with previous studies suggesting the release of more phenolic compound for the enzyme-treated samples compared to the untreated samples. The mechanistic explanation for this concept consist of the cell wall degradability feature for enzymes allowing the easy penetration of solvents into the matrix and releasing the bound phenolic compounds [13], [38]. The release of more phenolic of RA, FA, and cGA indicates that a substantial amount of these chia seed phenolic may exist in its bound form which need the disruption of the matrix in order to release the full amount of the compounds, using the viscozyme and amylase. Oliveria-Alves et al., [4] and Mitrovic et al., [7] explained the presence of a significant amount of bound phenolic in chia seeds consisting of cGA, RA, FA, caffeic acid etc. These phenolic remain as insoluble bound fraction and upon chemical hydrolysis along with the use of cell wall disrupting enzymes such as viscozyme and amylase, the phenolic are converted to its free form resulting an enhanced liberation from the matric [4], [7], [8]. This suggest a plausible mechanism for the enhanced recovery of cGA, RA, and FA from chia seeds samples using viscozyme and amylase enzyme.

The MEM, alike EM, yielded the highest phenolic amount for FA and RA. A strong synergistic phenomenon was observed for MEM resulting more phenolic yield compared to EM. The mixtures of protease, amylase, and viscozyme exhibited more synergy with enhanced phenolic release from chia seed matrix. The combination of enzymes and synergy observed reveals that the phenolic compounds are bound not only to the cell wall and polysaccharides but also to the membranous or proteinaceous structures. In such cases the cocktail of different enzymes such as protease, amylase, cellulase, and viscozyme is the more appropriate option to efficiently disrupt the cell wall, cell membrane, and protein structures to allow an easy exit for the phenolic compounds [15], [38]. This represent MEM a highly efficient method for obtaining phenolic-rich extracts from chia seeds for nutraceutical or food applications. Herein, different concentrations of enzymes at a fix temperature and time were studied, resulting a significantly better yield. Nevertheless, future work with a focus on multivariate factors (pH, time, temperatures, solid-solvent ratio) is needed to optimize the conditions for enzymatic hydrolysis. This may help select the best-fit model for enzymes in terms of solid: solvent ratio, effect of temperature and time to recover more yield for phenolic in chia seeds.

The statistical analysis of paired sample *t*-test exhibited significant differences (*P* < 0.05) for the use of enzyme EM and MEM vs the phenolic yield from chia seeds. The MEM resulted more phenolic yield compared to the EM suggesting the synergy concept and supporting the data observed. Likewise, the K-mean clustered the samples into different categories based on the nature of enzymes used vs the phenolic yield obtained in the study. The samples with more yield for QT and KF were observed in protease enzyme digested samples whereas, the samples with more amount for cGA, RA, and FA highlighted the use of amylase and viscozyme either alone or in combination for an enhanced yield. The data from the statistical models further supports the hypothesis and mechanisms discussed previously, for the use of the enzyme type with an enhanced effect on phenolic yield.

Chia seeds are well recognized for its anti-inflammatory, antioxidant, anti-hyperlipidemia, and cardio-protective effects. These are attributable to the presence of phenolic compounds such as RA, FA, cGA, QT, and KF. For instance: potent antioxidant and anti-inflammatory activities have been observed for RA and FA, mediated via ROS scavenging effect and NF-κB signals modulation [39], [40], cGA inhibits lipid peroxidation to improve lipid profile [41], QT and KF showed potent cardio-protective effects through improvement of the endothelial function and reducing the oxidative stress [42]. To understand the effect of temperature and time on the enzyme-effect on phenolic in chia seeds, a detailed kinetic study is proposed. A larger dataset focusing on the geographical origin variations of chia seeds, including factors such as soil composition, pH, temperature, fertilizers used, and post-harvest conditions, can help to better understand the composition and regional requirements necessary to produce high-quality chia seeds with an enriched profile.

The extraction method developed herein, in particular the enzymes-assisted extraction, with simultaneous quantification for the phenolic compounds resulted phenolic-enriched extract from chia seeds. This approach provide foundation for developing proper strategies for the preparation of chia-derived phenolic-enriched extract for antioxidant, anti-hypercholesteremic, and value-added functional foods products. Additionally, the validation of the extraction method on a large scale using the eleven different origin chia seeds samples for intra- and inter-variation of phenolic compounds may serve as a tool for the standardization and quality control of the chia seeds and its related herbal, dietary, and pharmaceutical products available in the market.

## 5 Conclusion

The study developed a successful extraction and quantification model for chia phenolic. The green solvent system of ACT: H_2_O (70: 30) with UA demonstrated a superior model for extraction whereas, UPLC-DAD showed a rapid and reliable analytical method for the simultaneous determination of phenolic: cGA, RA, FA, QT, and KF. The individual as well as the mixed-enzymes models exhibited a significant enhancement for these phenolic confirming the bound-form of the phenolic requiring enzymatic disruption for the cell wall. The large scale validation of the developed extraction method suggested a substantial variation for the phenolic in eleven different origin chia seeds samples indicating the need for standardization and quality evaluation to produce a standardized extract. Future studies should be directed toward combining phenolic profiling with extensive bioactivity assessment such as antioxidant activity using DPPH and FRAP assays, and biological models to correlate certain phenolic compounds with their bioactivity in chia seeds. The developed method offers the possibility of creating a robust model on the laboratory scale, which can be used for phenolic compounds' extraction and profiling from chia seeds. However, the optimization and validation of the process on the pilot scale will be needed to assess its industrial applications.

## 6 Authors contribution

RA (concept and study design); AA, MA, MR (write up for literature, introduction, and discussion); MAK, FA, MK, MAG (samples collection, processing, and extraction using ultrasonic, drying, filtration, and dilution of the samples for UPLC analysis) RA, MdA (method development and validation for UPLC-DAD analysis); RA, MA, AA, MdA (write up for material and methods, results, and discussion); RA, MR (statistical analysis); MAK, FA, MK, MAG (validation of the results and data); RA, MA, AA, MdA, MR (review/edit with final approval of the manuscript).

## 7 Consent to publish

The authors provided the consent to publish the data in the manuscript.

## 8 Availability of data

The data generated in provided in the form of tables and figures in the manuscript.

## Funding source

This research work was funded by Umm Al-Qura University, Saudi Arabia under grant number: 26UQU4280107GSSR01.

## CRediT authorship contribution statement

**Rizwan Ahmad:** . **Aljawharah Alqathama:** Writing – review & editing, Writing – original draft, Funding acquisition, Data curation. **Mohammed Aldholmi:** . **Mohammad Alkawi:** . **Faisal Alnaeem:** . **Majed Khan:** . **Mutaz Algarzai:** . **Mohd Amir:** . **Muhammad Riaz:** .

## Declaration of competing interest

The authors declare that they have no known competing financial interests or personal relationships that could have appeared to influence the work reported in this paper.
